# Distinct gamma oscillations in the distal dendritic fields of the dentate gyrus and the CA1 area of mouse hippocampus

**DOI:** 10.1007/s00429-017-1421-3

**Published:** 2017-04-08

**Authors:** Bálint Lasztóczi, Thomas Klausberger

**Affiliations:** Division of Cognitive Neurobiology, Centre for Brain Research, Medical University of Vienna, Spitalgasse 4, 1090 Vienna, Austria

**Keywords:** Hippocampus, Gamma oscillations, CA1, Dentate gyrus, Entorhinal cortex

## Abstract

The molecular layer of the dentate gyrus and the anatomically adjacent stratum lacunosum-moleculare of CA1 area, represent afferent areas at distinct levels of the hippocampal trisynaptic loop. Afferents to the dentate gyrus and CA1 area originate from different cell populations, including projection cells in entorhinal cortex layers two and three, respectively. To determine the organization of oscillatory activities along these terminal fields, we recorded local field potentials from multiple sites in the dentate gyrus and CA1 area of the awake mice, and localized gamma frequency (30–150 Hz) oscillations in different layers by means of current source density analysis. During theta oscillations, we observed different temporal and spectral organization of gamma oscillations in the dendritic layers of the dentate gyrus and CA1 area, with a sharp transition across the hippocampal fissure. In CA1 stratum lacunosum-moleculare, transient mid-frequency gamma oscillations (CA1-gamma_M_; 80 Hz) occurred on theta cycle peaks, while in the dentate gyrus, fast (DG-gamma_F_; 110 Hz), and slow (DG-gamma_S_; 40 Hz) gamma oscillations preferentially occurred on troughs of theta waves. Units in dentate gyrus, in contrast to units in CA1 pyramidal layer, phase-coupled to DG-gamma_F_, which was largely independent from CA1 fast gamma oscillations (CA1-gamma_F_) of similar frequency and timing. Spike timing of units recorded in either CA1 area or dentate gyrus were modulated by CA1-gamma_M_. Our experiments disclosed a set of gamma oscillations that differentially regulate neuronal activity in the dentate gyrus and CA1 area, and may allow flexible segregation and integration of information across different levels of hippocampal circuitry.

## Introduction

The hippocampus receives inputs from associative cortical areas, and is a key structure for spatial navigation and episodic memory (O’Keefe and Nadel 1978; Buzsaki and Moser 2013). The majority of its external afferents originate in the entorhinal cortex (EC), and reach the hippocampal formation via the temporoammonic (TA), and perforant pathways (PP), that terminate in anatomically adjacent areas, separated by the hippocampal fissure (Witter 2012). The PP is involved in contextual memory (Kitamura et al. 2015), and connects layer two (L2) of the EC to the dentate gyrus (DG; van Groen et al. 2003; Witter 2012). This pathway forms the very initial stage of information processing in hippocampal circuitry. By contrast, the TA, which is indispensable in temporal association memory (Suh et al. 2011), originates from the more upstream layer three (L3) of the EC. The TA innervates a more downstream level of the hippocampal trisynaptic loop via terminals located in the stratum lacunosum-moleculare of the CA1 area (van Groen et al. 2003; Witter 2012).

Based on their distinct theta phase preference, and spectral and spatial distributions, gamma frequency (30–150 Hz) oscillations in the CA1 area have been further classified as slow (30–60 Hz; CA1-gamma_S_), mid-frequency (50–100 Hz; CA1-gamma_M_), and fast (90–150 Hz; CA1-gamma_F_) oscillations (Belluscio et al. 2012; Scheffer-Teixeira et al. 2012; Schomburg et al. 2014; Lasztoczi and Klausberger 2016). In the CA1 area, different gamma oscillations are associated with pathways selectively terminating in different layers (Colgin et al. 2009; Scheffer-Teixeira et al. 2012; Schomburg et al. 2014; Lasztoczi and Klausberger 2014). For example, transient CA1-gamma_M_ oscillations appear strongest in the stratum lacunosum-moleculare, and preferentially occur on the peaks of theta oscillations (Scheffer-Teixeira et al. 2012; Schomburg et al. 2014; Lasztoczi and Klausberger 2016). Simultaneously, a similar oscillation in L3 of the medial EC (mEC) occurs, and pyramidal cells in L3 discharge (Schomburg et al. 2014; Mizuseki et al. 2009). Thus, CA1-gamma_M_ oscillations may result from concerted synaptic activity in the TA pathway (Schomburg et al. 2014; Yamamoto et al. 2014; Colgin et al. 2009; Lasztoczi and Klausberger 2014) and regulate the communication from EC L3 to area CA1 of the hippocampus (Schomburg et al. 2014; Lasztoczi and Klausberger 2016). Afferents projecting to the DG arise from a different set of parent cells, and DG gamma oscillations appear different from CA1-gamma_M_ (Scheffer-Teixeira et al. 2012). Axons of the PP originate from calbindin immunonegative, EC L2 stellate cells (Ray et al. 2014; Kitamura et al. 2014). Stellate cells, together with GABA*ergic* L2 basket cells, form a microcircuit capable of autonomously generating powerful theta-nested gamma oscillations independent of the L3, in vitro (Pastoll et al. 2013; Middleton et al. 2008; Couey et al. 2013). Interestingly, most mEC L2 projection cells, and hilar mossy cells that give rise to a second major excitatory pathway to the DG (Witter 2012; Scharfman, 2016), fire counter-phase to L3 cells, on the trough of theta waves in vivo (Mizuseki et al. 2009; Senzai and Buzsaki 2017). To test if CA1 and DG network operations are segregated by different temporal organizations of gamma oscillations, we recorded local field potentials (LFP) and calculated instantaneous current source density (CSD) in the dendritic layers of DG and CA1. In addition, we recorded spiking activity of CA1 and DG units and investigated their phase coupling to different gamma oscillations in head-restrained mice during movement.

## Materials and methods

All animal procedures were carried out under licences approved by the Austrian Ministry of Science and in accordance with the relevant regulations of the Medical University of Vienna. Adult male C57BL/6J mice were implanted with a plastic head plate under isoflurane anaesthesia (3–4% for induction and 1.5–2% for maintenance). After recovery (1–2 days), the animals were habituated to head restraint (additional 1–2 days), were water restricted (1 ml water/day), and trained to perform unidirectional runs in a 4 m long linear virtual reality maze (Phenosys), for a small water reward. Animals controlled the maze by rotating an air-supported styrofoam ball in all directions, of which only rotations along the long axis of the maze were registered. For craniotomy (9–129 days after head plate implantation), animals were briefly re-anaesthetized, a small cranial window was drilled above their right dorsal hippocampus (1.3 mm lateral and 1.9 mm caudal from the Bregma), the dura was removed, and the brain surface was sealed with silicone (Kwik-Sil, World Precision Instruments). At least 4 h were given as recovery before the first recordings were performed.

On recording days, mice were head-fixed in the apparatus, and the recording electrodes were inserted. To record LFP, we used a linear silicon probe with 16 recording sites at 50 µm spacing (Neuronexus), inserted 1.3 mm lateral and 1.7–2.0 mm caudal from the Bregma, with the dorso-ventral positioning guided by the profile of theta oscillations, sharp waves, and ripple oscillations. Spiking activity was recorded from the CA1 stratum pyramidale or stratum granulosum of the DG, using another silicon probe inserted in an 8°–10° angle, 300−600 µm away (Neuronexus; four shanks spaced at 150 µm with four contacts/shank in tetrode arrangement, or two shanks spaced at 200 µm distance with eight staggered contacts/shank; in final position the shank tips were 100–300 µm from the other silicon probe). After the recording locations were reached, the brain surface was covered (saline, mineral oil or wax), and additional 15–20 min was allowed for the electrode positions to stabilize. The exact position of the recording electrodes within the hippocampal formation was inferred post hoc, by comparing electrode tracks in histological analysis to the electrophysiological activity profiles (Lasztoczi and Klausberger 2014, 2016). Only experiments where the linear silicon probe spanned all the layers from CA1 stratum pyramidale to DG molecular layer, and at least three contacts were ventral from the hippocampal fissure, were included. Units were analysed only from shanks unequivocally positioned in CA1 stratum pyramidale or DG stratum granulosum. From nine experiments (in five animals), data on CA1 place cells and their coupling to CA1 gamma oscillations during place field traversals have been reported in an earlier publication (Lasztoczi and Klausberger 2016).

Signals from the silicon probes were pre-amplified (1×; RA16AC, Tucker-Davis), amplified (1000×), band-pass filtered (0.1–475 Hz for LFP, and 0.1–6000 Hz for unit recordings; Lynx-8 signal conditioners; Neuralynx), and digitized (2 kHz for LFP, and 20 kHz for unit recordings; Power1401mkII controlled by Spike2 software, Cambridge Electronic Design). All analyses were limited to periods with theta oscillations, which almost exclusively corresponded to periods of movement, despite no behavioural variables were considered during their definition. A single contact was selected (typically from CA1 stratum oriens or pyramidale), and theta periods were semi-automatically detected when the theta (5–12 Hz) to delta (2–4 Hz) power ratio exceeded 4 in the LFP signal, with period boundaries checked and adjusted manually if necessary (Lasztoczi and Klausberger 2016; Lapray et al. 2012). In all analyses, instantaneous theta phase was taken from stratum pyramidale or equivalent (stratum oriens), by linear interpolation between peaks (180°) and troughs (0° and 360°) detected in theta-filtered (5–12 Hz), and down-sampled (400 Hz) LFP trace.

Instantaneous CSD traces on contact *n* were derived from LFP traces of the linear silicon probe, by estimating the second spatial derivative at every sampled time point *t*, using the equation:

$${\text{CSD}}_{n,t} = - \frac{{{\text{LFP}}_{n - 1,t} - 2*{\text{LFP}}_{n,t} + {\text{LFP}}_{n + 1,t} }}{{\Delta z^{2} }},$$

where LFP_*n,t*_, LFP_*n*−*1*,*t*_, and LFP_*n*+*1,t*_ are the LFP samples at time *t*, from contacts *n* and the two neighbouring contacts, respectively, and Δ*z* is the spacing (50 µm; Lasztoczi and Klausberger 2016; Mitzdorf 1985; Bragin et al. 1995). In CSD traces sinks/sources are presented downwards/upwards. To analyse gamma oscillations, CSD traces were subjected to wavelet transformation (Lasztoczi and Klausberger 2016), with a complex Morlet wavelet (20–150 Hz; 53 logarithmically equidistant frequencies; wavelet parameters of 1 and 1.5; maximum sink at 0° and 360° and maximum source at 180°). Using complex wavelets allowed us to extract the instantaneous phase and amplitude values for each wavelet scale, at any time point. To analyse cross-frequency phase-amplitude coupling, amplitudes of CSD gamma oscillations were *Z*-scored over theta periods of the entire recording, averaged within 18° theta phase bins, and averaged across theta cycles. Modulation index spectra were generated by subtracting the minima from the maxima of these averages at each frequency (wavelet scale). For each gamma oscillation a frequency range was defined in a selected contact by identifying a local maximum of the modulation index, and extending the range until it decreased to either 80%, or a local minimum. Selected contacts were typically from stratum lacunosum-moleculare (CA1-gamma_M_), stratum radiatum (CA1-gamma_S_), stratum pyramidale (CA1-gamma_F_), and the DG molecular layer (DG-gamma_F_ and DG-gamma_S_). To quantify the theta modulation of gamma oscillations, average *Z*-scored amplitudes from the selected contact were averaged over the frequency ranges. Theta ranges for each gamma oscillation were defined as theta phases when these averages were positive. To calculate theta phase dependent phase coherence between CSD signals in contact pairs, in each tenth of a theta cycle, a time sample was drawn, and the corresponding instantaneous phase spectra were extracted in all contacts, and for the contact pairs phase difference spectra were generated. By repeating this procedure for all theta cycles in a recording, for each contact pair, each tenth of theta cycle and each frequency, we derived a phase-difference distribution and the corresponding statistics, such as the mean phase difference, the phase locking value (PLV; the mean vector length of angles; Lachaux et al. 1999), and an estimated Rayleigh’s *P* value. To quantify gamma phase coherence between two spatially distinct gamma oscillations, we averaged the PLV values from the phase coherence matrix of the contact pair relevant for those two oscillations, over the overlap in frequency and theta phase ranges.

Spikes were extracted and clustered from silicon probe recordings using standard procedures (Csicsvari et al. 2003a; Lasztoczi and Klausberger 2016; Royer et al. 2012; Rossant et al. 2016). Extracted spikes were sorted into clusters putatively originating from single units by first running an automatic clustering algorithm, KlustaKwik (Rossant et al. 2016; Harris et al. 2000), followed by manual and automatic refinement of the clusters in Klusters or KlustaViewa software (Hazan et al. 2006; Rossant et al. 2016). In experiments with silicon probe shanks with eight staggered contacts, we used the masked EM algorithm of the new version of KlustaKwik for both spike extraction and clustering (Rossant et al. 2016). Units were classified as putative principal cells (or putative GABA*ergic* cells), if they had an overall firing rate below (above) 3 Hz, a spike width at 90% of the peak amplitude above (below) 0.5 ms, and an event autocorrelogram value below 10 ms (above 15 ms). Event autocorrelogram value was calculated by taking the count-weighted average of offset times at which event autocorrelogram counts exceeded the mean count calculated over a 50 ms window. Other units remained unclassified. Modulation of spike timing by theta oscillations was tested in units with at least 20 spikes during theta oscillations. To analyse the modulation of spike timing by gamma oscillations, instantaneous wavelet transform spectra corresponding to unit spikes were extracted, and summarized with phase coupling statistics calculated in a spectral manner. Phase coupling for a particular gamma oscillation in a particular recording contact was considered significant, if we observed non-uniform (*P* < 0.05, Rayleigh test) phase distribution within the frequency range of gamma oscillation (individually defined for each experiment, see above) for at least half of the frequencies (scales). Phase coupling to gamma oscillations was tested only if at least 200 spikes occurred during theta oscillations. Phase coupling to a particular gamma oscillation in general was considered significant if it was significant in the contact used to define the frequency range of that oscillation (see above), and in the case of DG-gamma_F_, DG-gamma_S_, CA1-gamma_M_, and CA1-gamma_S_, in one additional designated contact, typically in the DG molecular layer and the CA1 stratum radiatum. To quantify coupling strength and mean phase to a particular gamma oscillation, *r* values for the most relevant contact were averaged over the respective frequency range and the mean phase was calculated over the scales with significant coupling within this range. Unless stated otherwise, data are presented as mean ± SD, or mean angle ± circular SD, as appropriate.

## Results

**Fig. 1:**
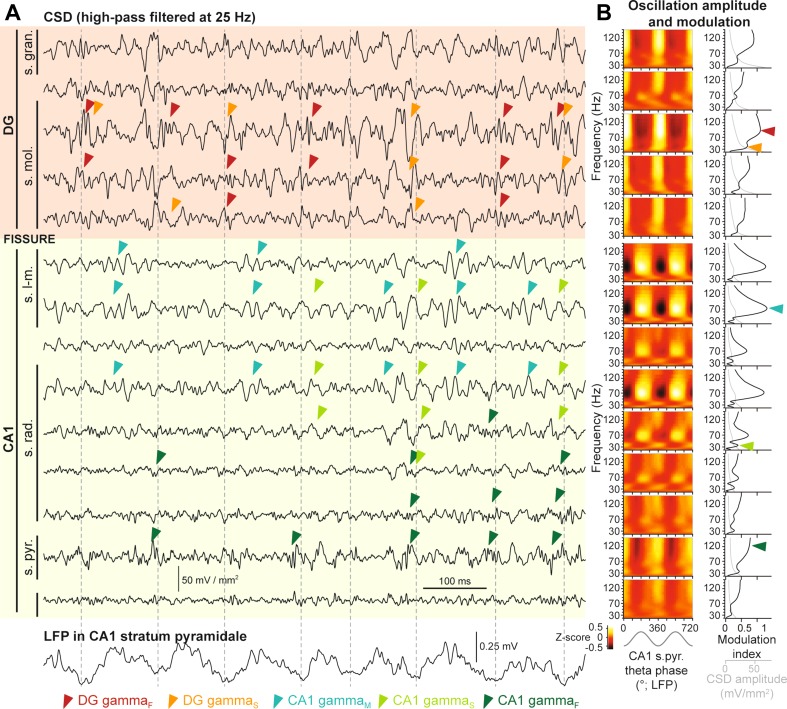
Gamma oscillations in the dentate gyrus and CA1 area of the hippocampus. **a** High-pass filtered (at 25 Hz) CSD traces calculated for silicon probe contacts located in different hippocampal input layers (source is upwards) and the corresponding LFP trace recorded from the CA1 pyramidal layer (*bottom trace*), during theta oscillations. Layers are indicated on the *left*. Theta troughs from the CA1 pyramidal layer LFP are marked by *vertical dotted lines*, for reference. *Coloured arrowheads* indicate instances of distinct gamma oscillations, as indicated. Dentate gyrus, and CA1 area are indicated by *red* and *green background colours*, respectively. **b** *Left*, mean amplitude of gamma oscillations (*Z*-score of CSD wavelet amplitude), plotted for each contact as a function of theta phase in CA1 pyramidal layer (18° bins, the theta cycle is duplicated for visualization), and gamma frequency (53 logarithmically equidistant wavelet scales between 20 and 150 Hz). *Right*, mean amplitude spectra (*grey*), and phase–amplitude modulation index spectra (*black*), of CSD from individual contacts. *Coloured arrowheads* indicate peak modulation index positions for various gamma oscillations in the most relevant contacts. Note the markedly different oscillatory dynamics across the fissure. *s. gran.* granule cell layer, *s.mol.* molecular layer, *s. l-m*. stratum lacunosum-moleculare, *s.rad.* stratum radiatum, *s. pyr.* stratum pyramidale, *CSD* current source density, *LFP* local field potential, *DG* dentate gyrus, *CA1* cornu ammonis area 1

**Fig. 2:**
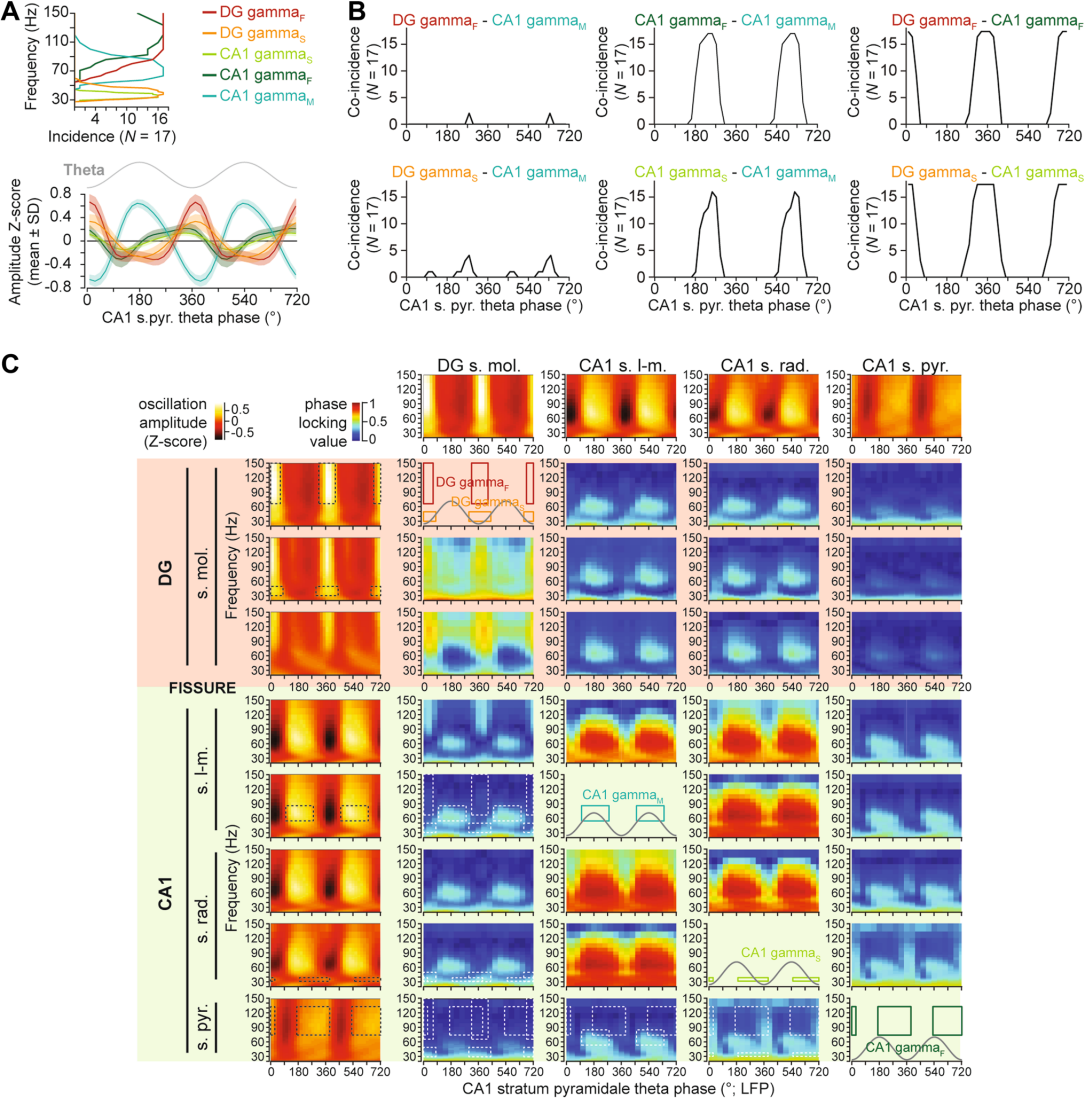
Co-occurrence and coherence of gamma oscillations in the dentate gyrus and CA1 area. **a** Frequency distribution (*upper plot*) and theta phase-amplitude modulation of different gamma oscillations. Theta phase here, and throughout the manuscript, was measured in the pyramidal cell layer of CA1 area. **b** Overlap in the occurrence (defined as a positive mean *Z* score) of different gamma oscillation pairs during the theta cycle. Note that the DG gamma_F_–CA1 gamma_F_ and DG gamma_S_–CA1 gamma_S_ oscillation pairs show substantial overlap in their frequency and theta phase distributions. **c** Phase coherence (measured as phase locking value; PLV) between oscillatory activities in contact pairs, displayed as a function of theta phase, and frequency. In the *top row*, and the *left column*, mean CSD oscillation amplitude *Z*-scores are plotted for selected contacts in stratum moleculare, stratum lacunosum-moleculare, stratum radiatum, and stratum pyramidale, as indicated. At the intercept of these, phase coherence between pairs of contacts (displayed as *colour-coded* phase locking value) is plotted as a function of theta phase (18° bins, the theta cycle is duplicated for visualization), and gamma frequency (53 logarithmically equidistant wavelet scales between 20 and 150 Hz). In plots at the intercept of a contact with itself, the theta phase and frequency ranges of different gamma oscillations prominent in the particular contact are displayed, for reference (also plotted as *black dotted lines* on the *left column* amplitude plots). *White dotted lines* indicate these same ranges on coherence plots, to indicate overlaps in frequency and theta phase ranges of different gamma oscillations

We recorded LFP, and calculated instantaneous CSD from multiple sites, in different layers of the CA1 area and the suprapyramidal blade of the DG, in the right dorsal hippocampus of head-fixed awake mice (*N* = 17 recording experiments from 9 animals). During theta oscillations, we observed strong and diverse oscillatory activity in the gamma frequency range (30–150 Hz), in CSD traces from all layers of the CA1 and DG (Fig. 1). Gamma oscillations were transient, and their occurrence depended on the phase of ongoing theta oscillation, and appeared strikingly different in the stratum lacunosum-moleculare of the CA1 area, and the molecular layer of the DG. In the CA1 stratum lacunosum-moleculare, high amplitude gamma oscillation transients were often observed on the peak of theta cycles, measured in the LFP of the CA1 pyramidal cell layer. These oscillations corresponded to CA1-gamma_M_ (Schomburg et al. 2014; Lasztoczi and Klausberger 2016), reached maximum amplitude at 193° ± 8°, and occupied a theta phase range between 117° and 279°, and a frequency range between 57 and 86 Hz (Figs. 1, 2a). By contrast, CSD traces in the DG displayed short bouts of faster oscillations, and longer transients of slower oscillations at the trough of theta cycles, counter-phase to CA1-gamma_M_ (Fig. 1). These oscillations have been mentioned before (Scheffer-Teixeira et al. 2012), here we characterize them in detail and we term them DG fast, and DG slow gamma oscillations (DG-gamma_F_ and DG-gamma_S_), respectively. DG-gamma_F_ and DG-gamma_S_ occupied a narrow range of the theta cycle around its trough (−63° to 63° and −63° to 81°, respectively; on average maximum amplitudes were at 8° ± 13° and 6° ± 21°, respectively; Figs. 1 and 2a). Despite the similar theta phase preference of DG-gamma_F_ and DG-gamma_S_, the modulation index spectra in DG showed biphasic frequency distributions, with the two oscillations occurring at frequency ranges 75–150, and 31–46 Hz, respectively (Figs. 1b, 2a). In the frequency domain, DG-gamma_S_ did not overlap with CA1-gamma_M_ but DG-gamma_F_ did (Fig. 2a). Within the theta cycle, however, DG-gamma_F_ and DG-gamma_S_ did not overlap with CA1-gamma_M_ (Figs. 1b, 2a, b).

In line with previous data (Schomburg et al. 2014; Lasztoczi and Klausberger 2016), in CA1 stratum radiatum and stratum pyramidale, we observed CA1-gamma_S_ (32–39 Hz) and CA1-gamma_F_ (92–150 Hz) with frequency ranges similar to DG-gamma_S_ and DG-gamma_F_ (Figs. 1, 2a). Both CA1-gamma_S_ and CA1-gamma_F_ were widely distributed (from −135° to 45°, and from −175° to 45°, respectively) on the descending phase of the theta waves recorded in the CA1 pyramidal cell layer (maximal amplitudes at 316° ± 17°, and 301° ± 11°, respectively; Figs. 1b, 2a), and thus overlapped substantially with the DG oscillations (Fig. 2b). This prompted us to analyse, if oscillatory phase of DG-gamma_S_–CA1-gamma_S_, and DG-gamma_F_–CA1-gamma_F_ oscillation pairs correlated in time. For quantification, we used the phase locking value (PLV; Lachaux et al. 1999; see Materials and Methods). Although often reaching significance thresholds (at *α* = 0.05, Rayleigh test), even when multiple comparisons were accounted for, the PLV values for DG-gamma_F_–CA1gamma_F_ pairs were small (0.061 ± 0.028, range 0.018–0.129), indicating that these two gamma oscillations were largely independent (Fig. 2c). By contrast, pairs of DG-gamma_s_–CA1-gamma_s_ showed stronger phase coherence (0.297 ± 0.066, range 0.134–0.386), suggesting that the two oscillations may partly reflect the same underlying oscillatory process (Fig. 2c). Unlike the DG oscillations, CA1-gamma_F_ and CA1-gamma_S_ showed substantial overlap with CA1-gamma_M_, during the early descending phase of the theta cycle (Figs. 1, 2b).

**Fig. 3:**
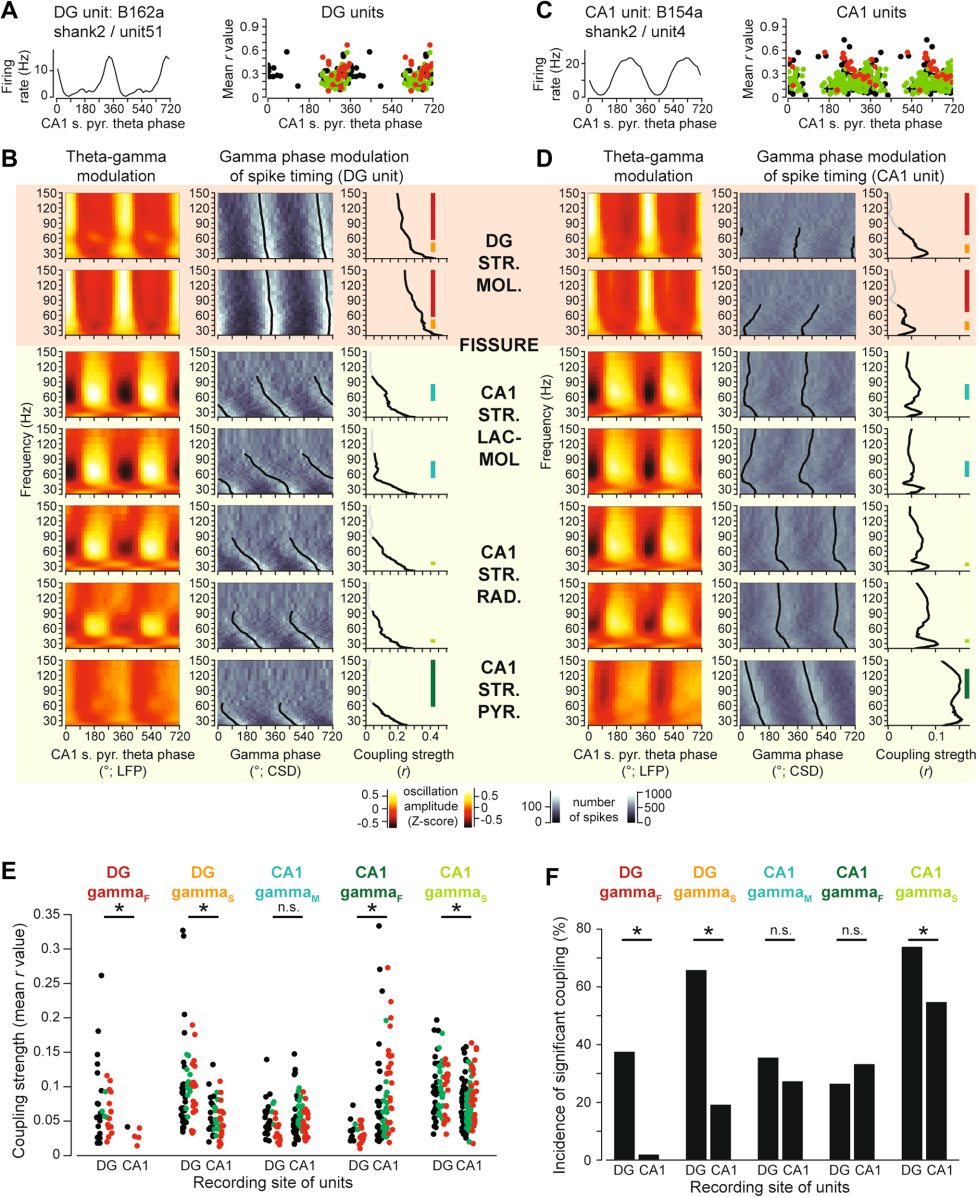
Phase modulation of spike-timing of units in the dentate gyrus and area CA1 by theta and different gamma oscillations. **a** *Left*, theta phase histogram of firing of one unclassified unit, recorded from the dentate gyrus. *Right*, mean theta phase of firing (abscissa), and modulation strength (mean *r* value, ordinate) of all significantly modulated putative principal cells (*green*), putative interneurons (*red*), and unclassified units (*black*), recorded from the dentate gyrus. **b** Spike-timing modulation by gamma oscillations during theta oscillations of one unit, recorded from the dentate gyrus (same unit as in **a**). *Left*, amplitude modulation of gamma oscillations by theta oscillations, in CSD traces from selected contacts in stratum moleculare of the dentate gyrus, and stratum lacunosum-moleculare, stratum radiatum, and stratum pyramidale of the CA1 area, as indicated. Middle, spike density (greyscale-coded) of the unit, plotted as a function of CSD gamma phase and frequency for the same contacts. The mean phase values for the frequencies significantly phase-modulating the spike timing are plotted in *black*. *Right*, modulation strength spectra (mean *r* values) for the unit, relative to gamma oscillations in different contacts (values at frequencies significantly modulating the spike-timing are plotted in *black*). Frequency ranges of the different gamma oscillations are displayed by *colour-coded bars* at the plots of most relevant contacts. **c** Same as in A for units recorded from the CA1 area. **d** Same as in **b**, for one unit recorded in the CA1 area (the same putative interneuron as in **c**). **e** Spike-timing modulation strength (mean *r* values) of units recorded in the dentate gyrus (DG) and the CA1 area, by different gamma oscillations. Only units with significantly non-uniform phase distribution are shown. Putative principal cells, interneurons, and unclassified units are plotted in *green*, *red*, and *black*, respectively. *Asterisks* denote significant differences by Mann-Whitney *U* test with Holm-Bonferroni correction procedure for multiple comparisons at a general *α* = 0.05. Note that CA1 units do not phase couple to DG-gamma_F_ and DG units are only weakly phase modulated by CA1-gamma_F_. **f** Percentage of DG and CA1 units with spike-timing significantly modulated by different gamma oscillations. *Asterisks* denote significant differences by the *χ* ^2^ test with Holm-Bonferroni correction procedure for multiple comparisons at a general *α* = 0.05

Along with gamma oscillations in the hippocampal formation, we recorded spikes of units from the granule cell layer of DG (*N* = 122 units, 44 putative principal cells, 21 putative interneurons, in 7 recording experiments from 3 mice), and the pyramidal layer of CA1 (*N* = 294 units, 161 putative pyramidal cells, 23 putative interneurons, in 10 recording experiments from 6 mice). Most DG (*N* = 112, 96%) and CA1 (*N* = 278, 95%) units were significantly (*P* < 0.05 with Rayleigh test) modulated by theta oscillations, and coupled with variable strength to the late descending (DG units) or descending (CA1 units) phase of the CA1 theta cycle (Fig. 3a, c).

Spectra of spike timing modulation by oscillation phase in the gamma frequency range showed a high degree of variability in different contacts, and across different units, even from the same hippocampal subfield. Importantly, DG-gamma_F_ entrained 37% (*N* = 37 of 99) of DG units with mean *r* of 0.072 ± 0.05 (range 0.018–0.261), but had only weak influence on the spike-timing (*r* 0.030 ± 0.012, range 0.014–0.042; *P* = 0.012 compared to DG units with Mann-Whitney *U* test) in a small subpopulation of CA1 units (*N* = 5 of 284, 1.8%; *P* = 1.6 × 10^−22^, *χ* ^2^ = 95, compared to DG units with *χ* ^2^ test; Fig. 3b, d–f). However, CA1-gamma_M_ phase-modulated the spike timing of CA1 and DG units to similar extent (*r* in CA1: 0.053 ± 0.025, range 0.017–0.148; *r* in DG: 0.048 ± 0.025, range 0.016–0.139; *P* = 0.3, Mann-Whitney *U* test), and in similar proportion of units (CA1: *N* = 77 of 284, 27%, DG: *N* = 35 of 99; 35%; *P* = 0.12; *χ* ^2^ = 2.4; *χ* ^2^ test; Fig. 3b, d–f). Despite CA1-gamma_F_ and DG-gamma_F_ showing little coherence, and CA1 units firing independent of DG-gamma_F_, DG units were modulated by CA1-gamma_F_ in similar proportion (*N* = 26 of 99, 26%; *P* = 0.21; *χ* ^2^ = 1.6; *χ* ^2^ test), yet substantially weaker (*r* 0.032 ± 0.015, range 0.010–0.073; *P* = 1.8 × 10^−6^, Mann-Whitney *U* test) than CA1 units (*N* = 94 of 284, 33%; *r* 0.078 ± 0.063, range 0.019–0.334; Fig. 3e, f). Phase of slow gamma oscillations, recorded in either DG or CA1, modulated the firing of more DG units than CA1 units (DG-gamma_S_, *N* = 65 of 99 DG units, 65%, and 54 of 284 CA1 units, 19%; *P* = 5.9 × 10^−18^; *χ* ^2^ = 75; *χ* ^2^ test; CA1-gamma_S_, *N* = 73 of 99 DG units, 74%, and 155 of 284 CA1 units, 55%; *P* = 0.00028; *χ* ^2^ = 13; *χ* ^2^ test). Also, DG units displayed stronger modulation than the CA1 units (DG-gamma_S_, *r* 0.098 ± 0.056 vs. 0.053 ± 0.025, ranges 0.033–0.327 and 0.014–0.132; *P* = 1.3 × 10^−8^, Mann-Whitney *U* test; CA1-gamma_S_, *r* 0.095 ± 0.038 vs. 0.074 ± 0.031, ranges 0.031–0.196 and 0.017–0.164; *P* = 6*10^−5^, Mann-Whitney *U* test; Fig. 3e, f). These data indicate that DG-gamma_F_ represents an oscillation independent of CA1-gamma_F_, while DG-gamma_S_ and CA1-gamma_S_ may, at least partly, reflect the same underlying oscillatory process.

## Discussion

Here we report and characterise a class of gamma oscillations expressed in the molecular layer of the DG. Using CSD analysis in different layers of the hippocampus, in awake head-fixed mice, we observed short transients of around 110 Hz oscillations localised to the recording sites positioned within the DG, and restricted to a narrow temporal window around the trough of theta oscillations. We termed these DG fast gamma oscillations (DG-gamma_F_). In the rodent hippocampus, multiple gamma oscillations have been described (Bragin et al. 1995; Csicsvari et al. 2003b; Colgin et al. 2009; Scheffer-Teixeira et al. 2012; Schomburg et al. 2014; Lasztoczi and Klausberger 2014, 2016). However, its high frequency, and preferred theta phase, distinguishes DG-gamma_F_ from CA1-gamma_S_ (frequency around 35 Hz) and CA1-gamma_M_ (occurring on theta peaks). Moreover, DG-gamma_F_ and CA1-gamma_F_ display little phase coherence and unlike CA1-gamma_F_, DG-gamma_F_ influence the spike-timing of DG units, but not CA1 units. These two lines of evidence suggest that DG-gamma_F_ are different also from CA1-gamma_F_, and thus represent a distinct hippocampal gamma oscillation, likely corresponding to the wide-band, fast gamma oscillations observed in LFP recordings from the rat DG (Scheffer-Teixeira et al. 2012). Extracellular potentials resulting from neuronal spikes have significant power in the gamma_F_ frequency range (Ray and Maunsell 2011; Belluscio et al. 2012). This raises the possibility that uncoordinated multiunit activity (axonal or somatic) may have contributed to both DG-gamma_F_ and CA1-gamma_F_ (Belluscio et al. 2012; Scheffer-Teixeira et al. 2013), and caused spurious phase modulation of spike-timing (Zanos et al. 2011; Scheffer-Teixeira et al. 2013). However, the amplitude of unitary extracellular spikes decreases by more than an order of magnitude over 100 µm (Henze et al. 2000; Schomburg et al. 2012), and we detected phase coupling of units to DG-gamma_F_ and CA1-gamma_F_ in electrodes typically >100 µm apart. Moreover, we observed coherent DG-gamma_F_ oscillations at multiple non-neighbouring, dendritic recording sites. Thus, gamma_F_, and in particular DG-gamma_F_, result from spiking and synaptic activities temporally coordinated over spatially extended populations of CA1 and DG cells (Schomburg et al. 2012). Spiking activity in CA1 stratum pyramidale and—more surprisingly—in DG as well, was phase-modulated by CA1-gamma_F_. Since DG-gamma_F_ and CA1-gamma_F_ are not coherent, this weak modulation in the DG may occur on descending phase of the theta cycle, when CA1-gamma_F_, but not DG-gamma_F_ are present, and may be mediated by a yet unknown common oscillatory input, or GABA*ergic* back-projections from CA1 to the DG (Katona et al. 2016; Klausberger et al. 2005; Fuentealba et al. 2010).

The temporal organization and frequency distribution of gamma oscillations is markedly different in the molecular layer of the DG and the stratum lacunosum-moleculare of the CA1 area, suggesting that network operations are segregated in these two structures. Glutamatergic innervation to the CA1 stratum lacunosum-moleculare arise from the pyramidal cells of mEC L3 (van Groen et al. 2003; Yamamoto et al. 2014; Witter 2012; Suh et al. 2011), where the frequency and theta phase preference of gamma oscillations mirror CA1-gamma_M_, suggesting that coordinated firing of L3 pyramidal cells on theta cycle peaks (Mizuseki et al. 2009) are transmitted via the TA, and generate CA1-gamma_M_ (Schomburg et al. 2014; Colgin et al. 2009; Yamamoto et al. 2014; Sun et al. 2015; Lasztoczi and Klausberger 2014, 2016). Units in the lateral EC (lEC) display weaker theta modulation (Deshmukh et al. 2010) and their contribution to CA1-gamma_M_ is poorly understood. On the other hand, the origins of DG gamma oscillations remain more elusive. Most glutamatergic terminals in the molecular layer of DG, where DG-gamma_F_ and DG-gamma_S_ were recorded, arise from the reelin-expressing, calbindin immunonegative principal cells of the mEC (stellate cells; Ray et al. 2014; Kitamura et al. 2014) and the lEC (Leitner et al. 2016), and the recurrent collaterals of hilar mossy cells (Scharfman 2016). Most L2 projection cells in the mEC (Mizuseki et al. 2009; Quilichini et al. 2010), and hilar mossy cells (Senzai and Buzsaki 2017), discharge in a narrow time window around the trough of theta oscillations, coincident with gamma oscillations in the DG (both fast and slow) and counter-phase to CA1 gamma_M_. Indeed, when rhythmically excited at theta frequency in vitro, the L2 microcircuit of the mEC (including stellate cells and mutually connected GABA*ergic* basket cells) generates transient synchronous gamma oscillations at every theta cycle, even when its L3 afferents are severed (Pastoll et al. 2013; Middleton et al. 2008; Couey et al. 2013), pointing to the PP as a potential source of DG-gamma_F_. Alternative explanations also exist (e.g. GABAergic networks within the DG generating gamma oscillations; Bragin et al. 1995; Pernia-Andrade and Jonas 2014), and further experimental work is needed to discriminate between these possibilities. Despite the lack of DG cell innervation by L3 (van Groen et al. 2003; Witter 2012), the spike timing of DG units was detectably correlated to the phase of CA1-gamma_M_. Potential mechanism for such entrainment include the GABAergic back-projection from CA1 to the DG (Katona et al. 2016; Klausberger et al. 2005; Fuentealba et al. 2010) or the entrainment of L2 network by L3 cells at theta peaks in the absence of its intrinsic rhythm as observed in vitro (Middleton et al. 2008).

The phase of DG-gamma_S_ and CA1-gamma_S_ were correlated during theta oscillations, and both slow gamma oscillations modulated the spike timing of units recorded from either the DG or the CA1 area. This indicates that DG-gamma_S_ is not independent from CA1-gamma_S_, and slow gamma oscillations may rather represent manifestations of the same underlying oscillatory process, that regulates information processing across the entire hippocampal formation, and perhaps beyond. Slow gamma oscillations have been linked to the communication between CA3 and CA1, and have been suggested to arise in the CA3 microcircuit (Csicsvari et al. 2003b; Colgin et al. 2009). In this case GABA*ergic* (Lasztoczi et al. 2011) and glutamatergic (Scharfman 2007) back-projections from CA3 to DG may mediate the entrainment of DG units by CA1-gamma_S_. Alternatively, slow gamma oscillations may originate upstream from CA3 in the DG or hilus, as originally proposed (Bragin et al. 1995). This latter scenario also offers a simple explanation to our finding that DG units couple stronger than CA1 units to both DG-gamma_S_ and CA1-gamma_S_. Indeed, it has been recently demonstrated that slow gamma oscillations persist when CA3 communication to CA1 area is blocked (Middleton and McHugh 2016) and Granger causality analysis indicated that slow gamma oscillations are imposed on CA3 pyramidal cells, by upstream microcircuits in DG (Hsiao et al. 2016). The preferred firing phase of both DG granule cells (Mizuseki et al. 2009; Senzai and Buzsaki 2017) and hilar mossy cells (Senzai and Buzsaki 2017) is consistent with their roles in generating DG-gamma_S_ and CA1-gamma_S_.

Multiple gamma oscillations are instrumental in regulating the communication along different extrinsic and intrinsic connections of the hippocampus (Colgin et al. 2009; Schomburg et al. 2014); yet experimental support on the actual roles of distinct gamma oscillations is sparse. Synchronization of CA1 pyramidal cells to CA1-gamma_M_ contributes to hippocampal network operations (Lasztoczi and Klausberger 2016; Yamamoto et al. 2014; Schomburg et al. 2014), and different gamma oscillations have been linked to different navigation strategies in rodents (Cabral et al. 2014). Our data indicates that the complexity and flexibility of animal cognition and behaviour may be supported by a diversity of gamma oscillations exceeding that previously thought, and that synaptic communication to different levels of hippocampal information processing is regulated by a set of distinct gamma oscillations.

## References

[CR1] Belluscio MA, Mizuseki K, Schmidt R, Kempter R, Buzsáki G (2012). Cross-frequency phase-phase coupling between theta and gamma oscillations in the hippocampus. J Neurosci.

[CR2] Bragin A, Jando G, Nadasdy Z, Hetke J, Wise K, Buzsaki G (1995). Gamma (40–100 Hz) oscillation in the hippocampus of the behaving rat. J Neurosci.

[CR3] Buzsaki G, Moser EI (2013). Memory, navigation and theta rhythm in the hippocampal-entorhinal system. Nat Neurosci.

[CR4] Cabral HO, Vinck M, Fouquet C, Pennartz CM, Rondi-Reig L, Battaglia FP (2014). Oscillatory dynamics and place field maps reflect hippocampal ensemble processing of sequence and place memory under NMDA receptor control. Neuron.

[CR5] Colgin LL, Denninger T, Fyhn M, Hafting T, Bonnevie T, Jensen O, Moser MB, Moser EI (2009). Frequency of gamma oscillations routes flow of information in the hippocampus. Nature.

[CR6] Couey JJ, Witoelar A, Zhang SJ, Zheng K, Ye J, Dunn B, Czajkowski R, Moser MB, Moser EI, Roudi Y, Witter MP (2013). Recurrent inhibitory circuitry as a mechanism for grid formation. Nat Neurosci.

[CR7] Csicsvari J, Henze DA, Jamieson B, Harris KD, Sirota A, Bartho P, Wise KD, Buzsaki G (2003). Massively parallel recording of unit and local field potentials with silicon-based electrodes. J Neurophysiol.

[CR8] Csicsvari J, Jamieson B, Wise KD, Buzsaki G (2003). Mechanisms of gamma oscillations in the hippocampus of the behaving rat. Neuron.

[CR9] Deshmukh SS, Yoganarasimha D, Voicu H, Knierim JJ (2010). Theta modulation in the medial and the lateral entorhinal cortices. J Neurophysiol.

[CR10] Fuentealba P, Klausberger T, Karayannis T, Suen WY, Huck J, Tomioka R, Rockland K, Capogna M, Studer M, Morales M, Somogyi P (2010). Expression of COUP-TFII nuclear receptor in restricted GABAergic neuronal populations in the adult rat hippocampus. J Neurosci.

[CR11] Harris KD, Henze DA, Csicsvari J, Hirase H, Buzsaki G (2000). Accuracy of tetrode spike separation as determined by simultaneous intracellular and extracellular measurements. J Neurophysiol.

[CR12] Hazan L, Zugaro M, Buzsaki G (2006). Klusters, NeuroScope, NDManager: a free software suite for neurophysiological data processing and visualization. J Neurosci Methods.

[CR13] Henze DA, Borhegyi Z, Csicsvari J, Mamiya A, Harris KD, Buzsaki G (2000). Intracellular features predicted by extracellular recordings in the hippocampus in vivo. J Neurophysiol.

[CR14] Hsiao YT, Zheng C, Colgin LL (2016). Slow gamma rhythms in CA3 are entrained by slow gamma activity in the dentate gyrus. J Neurophysiol.

[CR15] Katona L, Micklem B, Borhegyi Z, Swiejkowski DA, Valenti O, Viney TJ, Kotzadimitriou D, Klausberger T, Somogyi P (2016). Behavior-dependent activity patterns of GABAergic long-range projecting neurons in the rat hippocampus. Hippocampus.

[CR16] Kitamura T, Pignatelli M, Suh J, Kohara K, Yoshiki A, Abe K, Tonegawa S (2014). Island cells control temporal association memory. Science.

[CR17] Kitamura T, Sun C, Martin J, Kitch LJ, Schnitzer MJ, Tonegawa S (2015). Entorhinal cortical ocean cells encode specific contexts and drive context-specific fear memory. Neuron.

[CR18] Klausberger T, Marton L, O’Neill J, Huck J, Dalezios Y, Fuentealba P, Suen W, Papp E, Kaneko T, Watanabe M, Csicsvari J, Somogyi P (2005). Complementary roles of cholecystokinin- and parvalbumin-expressing GABAergic neurons in hippocampal network oscillations. J Neurosci.

[CR19] Lachaux JP, Rodriguez E, Martinerie J, Varela FJ (1999). Measuring phase synchrony in brain signals. Hum Brain Mapp.

[CR20] Lapray D, Lasztoczi B, Lagler M, Viney TJ, Katona L, Valenti O, Hartwich K, Borhegyi Z, Somogyi P, Klausberger T (2012). Behavior-dependent specialization of identified hippocampal interneurons. Nat Neurosci.

[CR21] Lasztoczi B, Klausberger T (2014). Layer-specific GABAergic control of distinct gamma oscillations in the CA1 hippocampus. Neuron.

[CR22] Lasztoczi B, Klausberger T (2016). Hippocampal place cells couple to three different gamma oscillations during place field traversal. Neuron.

[CR23] Lasztoczi B, Tukker JJ, Somogyi P, Klausberger T (2011). Terminal field and firing selectivity of cholecystokinin-expressing interneurons in the hippocampal CA3 area. J Neurosci.

[CR24] Leitner FC, Melzer S, Lutcke H, Pinna R, Seeburg PH, Helmchen F, Monyer H (2016). Spatially segregated feedforward and feedback neurons support differential odor processing in the lateral entorhinal cortex. Nat Neurosci.

[CR25] Middleton SJ, McHugh TJ (2016). Silencing CA3 disrupts temporal coding in the CA1 ensemble. Nat Neurosci.

[CR26] Middleton S, Jalics J, Kispersky T, Lebeau FE, Roopun AK, Kopell NJ, Whittington MA, Cunningham MO (2008). NMDA receptor-dependent switching between different gamma rhythm-generating microcircuits in entorhinal cortex. Proc Natl Acad Sci USA.

[CR27] Mitzdorf U (1985). Current source-density method and application in cat cerebral cortex: investigation of evoked potentials and EEG phenomena. Physiol Rev.

[CR28] Mizuseki K, Sirota A, Pastalkova E, Buzsaki G (2009). Theta oscillations provide temporal windows for local circuit computation in the entorhinal-hippocampal loop. Neuron.

[CR29] O’Keefe J, Nadel L (1978). The hippocampus as a cognitive map.

[CR30] Pastoll H, Solanka L, van Rossum MC, Nolan MF (2013). Feedback inhibition enables theta-nested gamma oscillations and grid firing fields. Neuron.

[CR31] Pernia-Andrade AJ, Jonas P (2014). Theta-gamma-modulated synaptic currents in hippocampal granule cells in vivo define a mechanism for network oscillations. Neuron.

[CR32] Quilichini P, Sirota A, Buzsáki G (2010). Intrinsic circuit organization and theta-gamma oscillation dynamics in the entorhinal cortex of the rat. J Neurosci.

[CR33] Ray S, Maunsell JH (2011). Different origins of gamma rhythm and high-gamma activity in macaque visual cortex. PLoS Biol.

[CR34] Ray S, Naumann R, Burgalossi A, Tang Q, Schmidt H, Brecht M (2014). Grid-layout and theta-modulation of layer 2 pyramidal neurons in medial entorhinal cortex. Science.

[CR35] Rossant C, Kadir SN, Goodman DF, Schulman J, Hunter ML, Saleem AB, Grosmark A, Belluscio M, Denfield GH, Ecker AS, Tolias AS, Solomon S, Buzsaki G, Carandini M, Harris KD (2016). Spike sorting for large, dense electrode arrays. Nat Neurosci.

[CR36] Royer S, Zemelman BV, Losonczy A, Kim J, Chance F, Magee JC, Buzsaki G (2012). Control of timing, rate and bursts of hippocampal place cells by dendritic and somatic inhibition. Nat Neurosci.

[CR37] Scharfman HE (2007). The CA3 “backprojection” to the dentate gyrus. Prog Brain Res.

[CR38] Scharfman HE (2016). The enigmatic mossy cell of the dentate gyrus. Nat Rev Neurosci.

[CR39] Scheffer-Teixeira R, Belchior H, Caixeta FV, Souza BC, Ribeiro S, Tort AB (2012). Theta phase modulates multiple layer-specific oscillations in the CA1 region. Cereb Cortex.

[CR40] Scheffer-Teixeira R, Belchior H, Leao RN, Ribeiro S, Tort AB (2013). On high-frequency field oscillations (>100 Hz) and the spectral leakage of spiking activity. J Neurosci.

[CR41] Schomburg EW, Anastassiou CA, Buzsaki G, Koch C (2012). The spiking component of oscillatory extracellular potentials in the rat hippocampus. J Neurosci.

[CR42] Schomburg EW, Fernandez-Ruiz A, Mizuseki K, Berenyi A, Anastassiou CA, Koch C, Buzsaki G (2014). Theta phase segregation of input-specific gamma patterns in entorhinal-hippocampal networks. Neuron.

[CR43] Senzai Y, Buzsaki G (2017). Physiological properties and behavioral correlates of hippocampal granule cells and mossy cells. Neuron.

[CR44] Suh J, Rivest AJ, Nakashiba T, Tominaga T, Tonegawa S (2011). Entorhinal cortex layer III input to the hippocampus is crucial for temporal association memory. Science.

[CR45] Sun C, Kitamura T, Yamamoto J, Martin J, Pignatelli M, Kitch LJ, Schnitzer MJ, Tonegawa S (2015). Distinct speed dependence of entorhinal island and ocean cells, including respective grid cells. Proc Natl Acad Sci USA.

[CR46] van Groen T, Miettinen P, Kadish I (2003). The entorhinal cortex of the mouse: organization of the projection to the hippocampal formation. Hippocampus.

[CR47] Witter M, Paxinos G, Puelles L (2012). Chapter 5—hippocampus A2—Watson, Charles. The mouse nervous system.

[CR48] Yamamoto J, Suh J, Takeuchi D, Tonegawa S (2014). Successful execution of working memory linked to synchronized high-frequency gamma oscillations. Cell.

[CR49] Zanos TP, Mineault PJ, Pack CC (2011). Removal of spurious correlations between spikes and local field potentials. J Neurophysiol.

